# The effect of early postpartum vitamin d and e supplementation on uterine after pain: a double-blind randomized clinical trial

**DOI:** 10.1186/s12905-025-04021-6

**Published:** 2025-11-18

**Authors:** SeyedehZahra HosseiniHaji, Roya Baghani, Mina Ghalenovi, Narjes Forouhar, Maryam Aradmehr

**Affiliations:** 1 Department of Midwifery, School of Nursing and Midwifery, Torbat Heydariyeh University of Medical Sciences, Torbat Heydarieh, Iran; 2 Department of Midwifery, School of Nursing and Midwifery, Shahroud University of Medical Sciences, Shahroud, Iran; 3 Department of Midwifery, School of Nursing and Midwifery, Sabzevar University of Medical Sciences, Sabzevar, Iran; 4 Faculty of Medicine, Sabzevar University of Medical Sciences, Sabzevar, Iran

**Keywords:** Postpartum after pain, Vitamin e, Vitamin d, Randomized clinical trial, Pain management, Multiparous women

## Abstract

**Background:**

Uterine after pain is a common yet under-addressed postpartum complaint. This study aimed to investigate whether early postpartum supplementation with vitamins D and E could reduce the intensity and duration of uterine after pain in multiparous women.

**Methods:**

This double-blind, parallel-group randomized clinical trial was conducted between October and December 2022 at Shahid Mobini Hospital in Sabzevar, Iran. One hundred eligible multiparous women were randomly assigned to receive either eight capsules containing 100 mg of vitamin E and 400 IU of vitamin D over the first 48 h postpartum or matched placebo capsules. Pain intensity was assessed via the visual analog scale (VAS), beginning 2 h after delivery and subsequently at 6-hour intervals across eight time points. The duration of pain was measured via the Cox Menstrual Symptom Scale (CMSS). Data analysis was performed via SPSS version 16, with statistical significance set at *P* < 0.05.

**Results:**

No significant differences in pain intensity or duration at baseline were detected between the groups (*P* = 0.58 and *P* = 0.06, respectively). From the third time point onward, participants in the intervention group reported significantly lower pain intensity than did those in the placebo group (*P* < 0.001). A similar trend was observed for pain duration, with a statistically significant reduction starting from the second time point in the intervention group (*P* < 0.001).

**Conclusion:**

Supplementation with vitamins E and D during the early postpartum period may serve as a safe, effective, and accessible strategy for reducing the intensity and duration of uterine after pain in multiparous women. These findings support the integration of targeted micronutrient therapy into postpartum pain management protocols.

**Trial registration:**

Iranian Registry of Clinical Trials (IRCT): IRCT20220313054270N1.

## Highlights

Uterine after pain remains a challenging postpartum symptom with limited treatment options.

Combined supplementation with vitamins E and D significantly reduced both pain intensity and duration.

The effects became significant after the second to third doses were given postpartum.

The findings suggest a safe, low-cost, accessible adjunct to standard postpartum care.

## Introduction

Pregnancy and childbirth significantly impact women’s physical and emotional health and are considered critical indicators of a nation’s overall healthcare status. Following delivery, women often experience a range of pain conditions, including incisional pain after cesarean section, perineal discomfort due to episiotomy during vaginal birth, headaches following spinal anesthesia, and general postpartum uterine pain [1]. Among these, postpartum uterine pain—particularly after vaginal delivery—is one of the most prevalent and distressing complaints in maternity care [2]. The intensity of this pain ranges from mild cramping, similar to menstrual discomfort, to severe pain that can exceed labor pain in some cases [3]. Typically, such pain lasts for 3–4 days post-delivery; however, in some instances, it may persist for up to a week, with multiparous women being more frequently affected [4]. Postpartum pain is primarily attributed to uterine contractions during the involution process, triggered by the release of biochemical mediators such as prostaglandins, bradykinin, leukotrienes, serotonin, and lactic acid. Among these, prostaglandins are considered the central agents responsible for inducing uterine contractions and subsequent pain [2, 5, 6]. Reports indicate that the prevalence of postpartum pain in multiparous women reaches 77% [2, 3, 7, 8]. Unmanaged postpartum pain can lead to complications such as sleep disturbances, mood disorders, appetite loss, and difficulties in newborn care, underscoring the need for effective pain management strategies. Conventional treatments include pharmacological approaches—mainly oral analgesics such as ibuprofen, acetaminophen, and mefenamic acid—which are widely used in maternity settings [9]. While effective, these medications can cause adverse effects, including gastrointestinal discomfort, nausea, dizziness, and, in rare cases, more severe reactions, such as seizures [10]. Given the potential drawbacks of conventional analgesics, there is increasing global interest in alternative and complementary treatments [2]. Herbal remedies, traditional therapies, and nutritional supplements have gained popularity because of their safety profile, accessibility, and lower incidence of side effects [5, 11, 12]. According to the World Health Organization, a growing portion of the global population is turning to traditional medicine as a primary or complementary form of healthcare [13]. Among emerging non pharmacological interventions, vitamins D and E have garnered attention for their potential analgesic effects [11, 14]. Vitamin D is known to modulate inflammatory responses and reduce prostaglandin synthesis, mechanisms that are implicated in both menstrual and postpartum pain. Moreover, vitamin D receptors have been identified in reproductive tissues, including the endometrium, supporting their role in reproductive health [15–17]. Similarly, vitamin E exhibits anti-inflammatory properties by inhibiting the cyclooxygenase pathway, reducing prostaglandin production, and increasing the activity of endogenous opioids. Clinical trials have shown that vitamin E supplementation can alleviate the intensity and duration of dysmenorrhea, suggesting its potential utility in postpartum pain management as well [14]. Dadkhah et al. demonstrated that combined supplementation with vitamin E (100 mg) and vitamin D (200 mg) effectively reduces the severity of dysmenorrhea and premenstrual syndrome, with no reported adverse effects. Given their physiological role in inhibiting prostaglandin synthesis and their documented benefits in alleviating smooth muscle pain, these vitamins may offer promising alternatives for managing postpartum discomfort [18]. Considering the clinical importance of postpartum pain and its adverse effects on maternal well-being, self-care, infant bonding, and breastfeeding, exploring non pharmacological, safe, and accessible interventions is essential. Vitamin E and D supplements are known for their affordability, safety, widespread availability, and compatibility with lactation, making them suitable candidates for postpartum pain management [5, 15, 17, 19]. Therefore, the objective of the present study was to evaluate the effect of combined vitamin E and vitamin D supplementation on the severity of postpartum uterine pain.

## Methodology

This double-blind randomized clinical trial was conducted by the research team at Sabzevar University of Medical Sciences, Iran, from 2024 to 2025. The study population consisted of 100 postpartum mothers admitted to the postnatal care unit at Shahidan Mobini Hospital, affiliated with Sabzevar University of Medical Sciences.

### Sample size calculation

The sample size was calculated based on a previous randomized controlled trial by Ziaei et al. [20], which evaluated the effect of vitamin E on pain severity and duration. Using the reported effect size from that study (Cohen’s d ≈ 0.63), with a significance level of 5% (α = 0.05) and power of 80%, the minimum required sample size was determined as 45 participants per group. To account for potential attrition, this was increased to 50 participants per group, for a total of 100 participants in the study.

### Randomization and blinding

Participants were evaluated within 48 h postpartum and allocated into two groups—intervention and control—based on the day of hospital admission. Randomization was carried out using sealed envelopes, with allocation determined by the day of the week: mothers admitted on Saturdays, Mondays, and Wednesdays were assigned to Group A (intervention), while those admitted on Sundays, Tuesdays, and Thursdays were assigned to Group B (control). This systematic allocation method, while not purely random, was chosen to reduce allocation bias; however, the possibility of selection bias is acknowledged as a limitation. Both participants and data analysts were blinded to group allocation.

### Intervention

The intervention group received supplementation starting 2 h after delivery, administered every 8 h for a total of eight doses during the study period. Each sealed packet contained eight coated tablets of vitamin E (100 IU each) and eight tablets of vitamin D (400 IU each). The control group received identical-appearing placebo capsules on the same schedule.

### Inclusion and exclusion criteria

Eligible participants were mothers aged 18–35 years, who had spontaneous vaginal delivery at 37–42 weeks’ gestation, without epidural or spinal anesthesia, and no third- or fourth-degree perineal tears. Additional criteria included spontaneous placental and membrane expulsion, prim parity or second delivery, ability to read and write, exclusive breastfeeding, and experiencing moderate to severe postpartum pain (VAS ≥ 4). Participants with narcotic analgesic use within four hours prior to delivery, substance abuse, chronic medical conditions, previous abdominal or pelvic surgery, symptoms or diagnosis of vitamin D deficiency, or current vitamin D3 supplementation were excluded. Participants who developed severe postpartum complications, used other analgesics except mefenamic acid, or withdrew were excluded from analysis.

### Data collection and outcome measures

Demographic and obstetric data were collected via structured questionnaires. Pain intensity was assessed using the McGill Pain Questionnaire (MPQ) and a 10-cm Visual Analog Scale (VAS), with scores categorized as mild [1–3], moderate [4–6], or severe [7–10]. Only participants with moderate to severe pain (VAS ≥ 4) were included. Pain duration was assessed using the Cox Menstrual Symptom Scale (CMSS). Adverse effects related to supplementation were actively monitored via a dedicated questionnaire; participants were instructed to report any symptoms such as allergic reactions, nausea, vomiting, or dizziness for evaluation.

### Data handling and missing data

Missing data were minimized through close monitoring during data collection; any missing values were handled using appropriate statistical methods (e.g., last observation carried forward), as detailed in the statistical analysis section.

### Data collection timeline

Pain intensity was measured at nine time points:

T1: Two hours post-delivery (prior to supplement administration).

T2–T9: At six-hour intervals after each subsequent dose of supplement or placebo.

If inadequate pain relief was reported within one hour after intervention, a.

250 mg capsule of mefenamic acid was administered, and analgesic consumption was recorded.

### Ethical considerations

The study protocol was approved by the Institutional Ethics Committee of Sabzevar University of Medical Sciences. Written informed consent was obtained from all participants prior to enrollment.

## Results

**Fig. 1:**
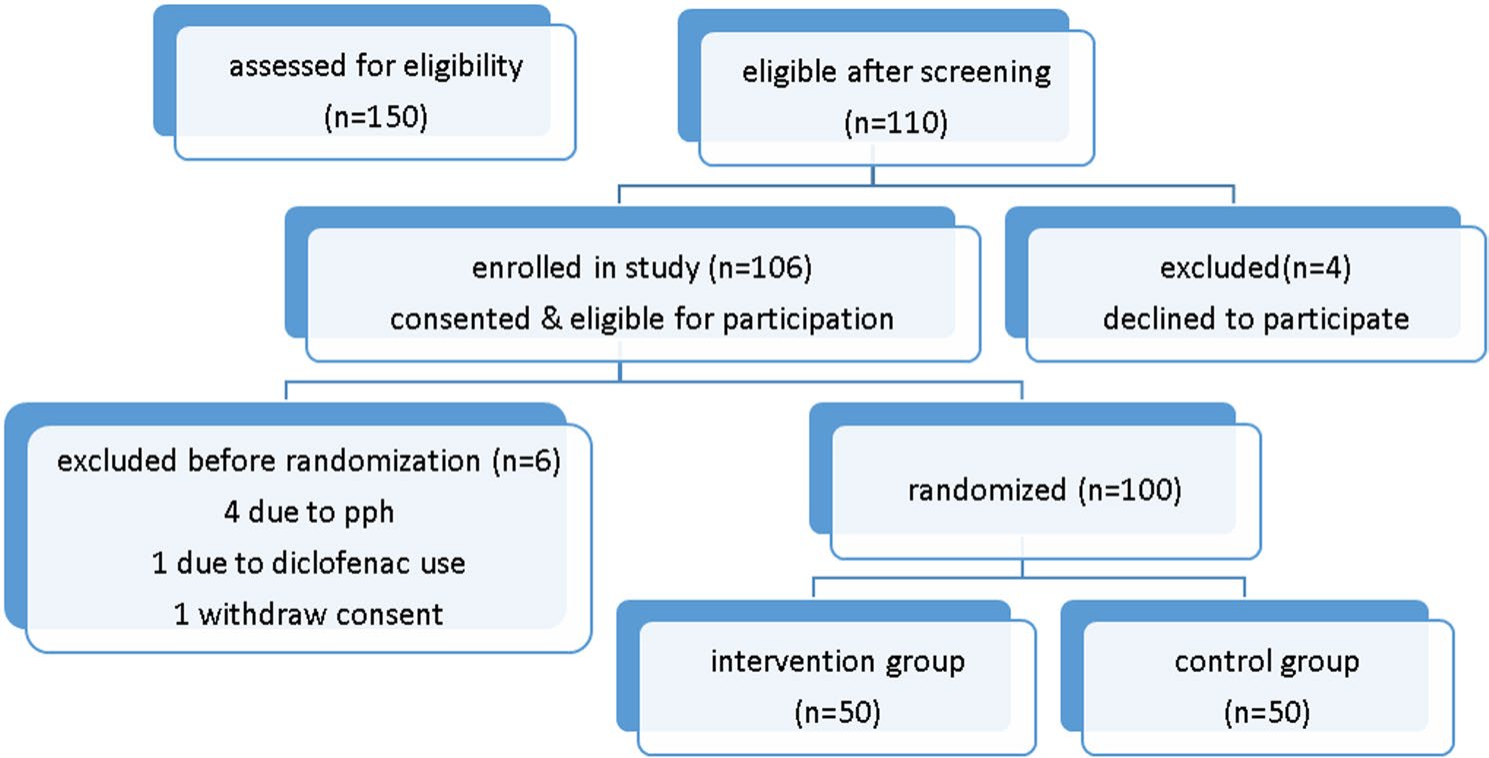
CONSORT flow diagram of participant recruitment and allocation

Among the 150 postpartum mothers initially assessed, 110 met the eligibility criteria on the basis of the screening questionnaire. Four individuals declined participation. Of the 106 mothers who satisfied the inclusion criteria, four were excluded because of increased postpartum hemorrhage, and one was excluded because of the use of a diclofenac suppository. Additionally, one participant voluntarily withdrew from the study. Ultimately, 100 mothers were randomized, with data from 50 participants in each group included in the final analysis (Fig. 1).

At baseline (T1), no statistically significant differences were observed between the intervention and control groups in any of the measured variables (Table 1). Pain intensity was assessed at baseline and at seven subsequent time points following the intervention. Both groups showed a progressive decrease in pain scores over time; however, the reduction was significantly greater in the vitamin-supplemented group than in the placebo group (Table 2). The duration of uterine cramps declined significantly across all time points in both groups. However, the difference between groups was not consistently significant, with the control group showing slightly greater reductions at several time points (Table 3). Furthermore, a significant decrease in mefenamic acid consumption was observed in the intervention group relative to the control group, corresponding with a greater reduction in reported pain scores (Table 4). No statistically significant differences were found between the groups regarding age or parity at baseline. In terms of baseline pain intensity, 74% of participants in the intervention group and 78% in the placebo group reported moderate pain, whereas 26% and 22%, respectively, reported severe pain. This difference was not statistically significant (*p* = 0.64) (Table 1).

**Table 1:** Comparison of patient characteristics before the intervention

Variables	Vit E and D (*n* = 50)	Placebo (*n* = 50)	*P* value
age (years)	31.60 (1.1)	30.57 (1.3)	0.07*
Parity (number)	1.6(0.3)	1.5(0.2)	0.053*
Pain score			
3.1- 6 (moderate)	37 (74)	39 (78)	0.64**
6.1–10 (severe)	13 (26)	11 (22)	0.64**

* t test ** Chi-square

**Table 2:** Comparison of mean pain intensity (± SD) between placebo and vitamin E + D groups at baseline and after each dose

	Vit E and D (*n* = 50)	Placebo (*n* = 50)	*P* value
Before the intervention (t1)	7.26 (0.664)	7.16 (1.072)	0.58*
6 h after first dose (t2)	6.17 (0.352)	6.36 (1.136)	0.26*
6 h after second dose (t3)	5.13 (0.334)	5.15 (1.342)	0.92*
6 h after third dose (t4)	4.17 (0.964)	5.33 (1.172)	*P* < 0.001*
6 h after fourth dose (t5)	3.17 (0.324)	4.60 (0.231)	*P* < 0.001*
6 h after fifth dose (t6)	2.10 (0.327)	4.15 (1.238)	*P* < 0.001*
6 h after sixth dose (t7)	1.84 (1.023)	3.65 (0.321)	*P* < 0.001*
6 h after seventh dose (t8)	1.10 (1.349)	2.25 (0.128)	*P* < 0.001*

*t test

**Table 3:** Comparison of pain duration before intervention and at seven time points post intervention

	Vit E and D (*n* = 50)	Placebo (*n* = 50)	*P* value
Before the intervention (t1)	36.60 (2.1)	35.57 (1.5)	0.06*
6 h after first dose (t2)	25.10 (3.352)	26.11(3.136)	0.12*
6 h after second dose (t3)	23.51(0.334)	24.23(1.342)	*P* < 0.001*
6 h after third dose (t4)	18.31(0.964)	16.12(1.172)	*P* < 0.001*
6 h after fourth dose (t5)	17.23(0.324)	15.23(0.231)	*P* < 0.001*
6 h after fifth dose (t6)	14.25(0.327)	13.32(1.238)	*P* < 0.001*
6 h after sixth dose (t7)	13.15(1.023)	12.20(0.321)	*P* < 0.001*
6 h after seventh dose (t8)	12.15(1.349)	11.20(0.128)	*P* < 0.001*

*t test

**Table 4:** Comparison of mefenamic acid consumption (mg/day)

Time Point	Placebo (Mean ± SD, mg)	Vit E and D (Mean ± SD, mg)	*P* value
Before the intervention (T1)	500 ± 0	500 ± 0	–
6 h after first dose (T2)	480 ± 20	440 ± 25	0.05
6 h after second dose (T3)	460 ± 30	400 ± 20	0.01
6 h after third dose (T4)	430 ± 25	350 ± 30	0.001
6 h after fourth dose (T5)	410 ± 28	300 ± 25	< 0.001
6 h after fifth dose (T6)	390 ± 30	250 ± 20	< 0.001
6 h after sixth dose (T7)	370 ± 35	200 ± 18	< 0.001
6 h after seventh dose (T8)	350 ± 38	150 ± 15	< 0.001

Statistical analysis was performed using independent t-test.

While no significant difference in mean pain duration was observed between groups at baseline (*p* = 0.06) or six hours after the first dose (T2) (vitamin group: 25.10 ± 3.35; placebo group: 26.11 ± 3.14; *p* = 0.12), statistically significant differences emerged from the time of the second dose (T3) onward (*p* < 0.001 at all subsequent time points). Both groups exhibited a downward trend in pain duration; however, the reduction was significantly more pronounced in the vitamin-supplemented group.

Collectively, these findings demonstrate that, compared with placebo, supplementation with vitamins E and D significantly reduced both pain intensity and duration, as well as analgesic use, particularly from the second dose of the intervention onward.

## Discussion

This study demonstrated that combined supplementation with vitamins E and D was associated with a significant reduction in both the intensity and duration of postpartum pain. Although pain levels decreased over time in both groups—likely due to natural postpartum recovery—the intervention group showed a statistically significant greater reduction starting after the second dose, indicating a potential cumulative or time-dependent effect of the supplementation [20]. The analgesic properties of vitamin E observed align with previous findings in primary dysmenorrhea, where it reduces pain by inhibiting arachidonic acid release and prostaglandin synthesis through antioxidant mechanisms [21]. This provides a plausible explanation for its effect on postpartum uterine pain, which similarly involves prostaglandin-mediated pathways. Consistent with Vilvapriya et al. (2018) [22], who reported pain reduction over three menstrual cycles, these results support the hypothesis that vitamin E influences uterine smooth muscle contractions. Additionally, plant-based interventions such as Triticum sativum, rich in vitamin E, have demonstrated beneficial effects on postpartum pain without adverse outcomes [23].

A distinctive feature of this trial was the combined administration of vitamins E and D. Given vitamin D’s known anti-inflammatory and immunomodulatory properties, co administration may enhance analgesic efficacy, although the possibility of synergistic effects warrants further exploration. The delayed onset of a statistically significant effect highlights the importance of investigating optimal dosing schedules, absorption kinetics, and pharmacodynamics.

### Practical implications

The supplementation regimen (vitamin E 100 IU + vitamin D 400 IU, started 2 h postpartum every 8 h for eight doses) was safe, affordable, and feasible in postpartum care. This approach could be particularly useful in low-resource settings or where reducing NSAID use is desired. Effective pain control may improve maternal mobility, sleep, mood, breastfeeding, and mother-infant bonding, while potentially lowering pharmacologic analgesic use. Recent reviews support nutritional and complementary methods for postpartum pain management but highlight the need for more high-quality trials [24, 25].

### Limitations and future directions

The current study’s 48-hour follow-up period hinders evaluation of longer-term outcomes such as functional recovery, sustained pain relief, psychological well-being, and any delayed adverse effects. The combined supplementation design prevents distinguishing the independent effects of vitamins E and D, and therefore cannot confirm whether synergistic mechanisms are at play.

Unmeasured confounders—such as maternal mental health, sleep quality, nutritional status, and prior pain experience—could influence pain perception and analgesic requirements, which the study did not account for systematically. Additionally, the absence of biochemical confirmation (e.g., serum 25‑OH‑D and α‑tocopherol levels) limits understanding of actual absorption, adherence, and dose–response relationships.

Recent reviews of vitamin D in the perinatal period indicate potential mood-related benefits, particularly for depressive symptoms, though current RCTs remain limited and of variable quality. This further underscores the importance of integrating mental health assessments and longer-term follow-up in future studies.

### Future investigations should consider

Factorial trial designs (e.g., separate arms for vitamin E, vitamin D, combination, and placebo) to clarify independent versus synergistic effects.

Biochemical monitoring of serum vitamin levels to confirm biologic uptake and explore dose–effect relationships.

Extended follow-up (ideally beyond the immediate postpartum, e.g., up to 6–12 weeks or longer) to assess functional recovery, mood, breastfeeding success, and safety.

Larger, well-powered samples, possibly stratified by population characteristics (e.g., nutritional status, baseline vitamin levels, mode of delivery).

Direct comparison with standard analgesics in scheduled dosing designs to evaluate relative efficacy, safety, and feasibility.

## Conclusion

In conclusion, this study provides preliminary evidence that co supplementation with vitamins E and D may offer a safe, accessible, and effective strategy for managing postpartum uterine pain. Further research with longer follow-up periods, diverse dosing regimens, and biochemical assessments is necessary to clarify the underlying mechanisms and confirm the sustained clinical benefits.

## Data Availability

The datasets used or analyzed during the current study are available from the corresponding author on reasonable request.

## Abbreviations

VAS: Visual analog scale
IU: International units
SPSS: Statistical package for the social sciences
